# Aspirin Use May Increase Atrial Fibrillation Risk: A Mendelian Randomization Analysis

**DOI:** 10.1155/ijog/6124554

**Published:** 2026-11-15

**Authors:** Gang Fan

**Affiliations:** ^1^ Department of Cardiology Xianyang Central Hospital Affiliated to Shaanxi University of Chinese Medicine Xianyang Shaanxi China

**Keywords:** aspirin use, atrial fibrillation, Mendelian randomization

## Abstract

**Purpose:**

The relationship between aspirin use and atrial fibrillation (AF) remains uncertain. This study is aimed at determining the causal association of aspirin use and AF risk by Mendelian randomization (MR) analysis.

**Methods:**

Single nucleotide polymorphisms (SNPs) for aspirin use and AF were obtained from genome‐wide association studies (GWAS). The inverse variance weighted (IVW) was used as the primary MR analysis method. In addition, I performed Cochran′s Q, MR‐Egger′s intercept, leave‐one‐out, MR‐PRESSO, and Radial MR to evaluate heterogeneity, horizontal pleiotropy, sensitivity, and reliability of the results.

**Results:**

Finally, 42 independent SNPs were selected as instrumental variables (IVs). The findings revealed that genetically proxied aspirin use elevated AF risk (odds ratio [OR]_IVW_ = 1.96 [95% confidence interval (CI) 1.10, 3.47], *p* = 0.021) in European population. The subsequent analysis confirmed the validity of the primary MR results. Cochran′s Q test (*p* > 0.05), MR‐Egger′s intercept (*p* > 0.05), MR‐PRESSO global test (*p* > 0.05), and Radial MR (*p* > 0.05).

**Conclusion:**

This MR study provides evidence suggesting a potential causal association between aspirin use and an increased risk of AF. However, given the inherent limitations of the MR design, these findings should be interpreted with caution. Future large‐scale randomized controlled trials (RCTs) with long‐term follow‐up are warranted to confirm and extend these observations.

## 1. Introduction

Atrial fibrillation (AF) is the most common arrhythmia, causing significant morbidity and mortality worldwide [1]. A cross‐sectional epidemiological study revealed that the population prevalence of AF in China was 1.6%, with higher percentages observed among the elderly [2]. Based on the findings of the 2021 Global Burden of Disease (GBD) study, AF impacted 52.55 million people globally, marking a 137% rise since 1990 [3]. The pathophysiology of AF encompasses a multifaceted array of factors, including atrial fibrosis and enlargement [4], electrophysiology anomalies [5], inflammation [6], and genetic susceptibility [7]. It is well established that numerous clinical risk factors contribute to the development of AF, including smoking [8], obesity [9], heart failure [10], and insomnia [11]. The treatment of AF mainly involves the modification of risk factors, the control of rate and rhythm, and the administration of anticoagulation therapy.

Aspirin was first introduced for its antipyretic, analgesic, and anti‐inflammatory properties, and went on to serve as a cornerstone of ischemic heart disease treatment for approximately 100 years [12]. Nevertheless, concerns have been articulated in relation to the routine prescription of aspirin for the principal prevention of cardiovascular disease [13, 14]. Moreover, the 2021 ESC and 2022 USPSTF guidelines did not advocate for aspirin to be used as a primary prevention treatment, particularly in elderly individuals anymore [15, 16]. A recent observational study indicated that the administration of aspirin is associated with an increased incidence of readmission due to heart failure (hazard ratio [HR] = 1.25; 95% CI: 1.17, 1.33) [17]. Additionally, a Mendelian randomization (MR) study revealed a positive causal relationship between aspirin and heart failure risk (OR = 1.09 [95% CI 1.02, 1.16]) [18]. The evidence regarding the administration of aspirin and the subsequent incidence of AF is relatively limited. A cohort study from the Physicians′ Health Study (PHS) showed that aspirin use for more than 180 days increased the risk of AF in the crude model (HR = 1.21; 95% CI: 1.10, 1.34), but there was no statistical significance after adjustment for age, BMI, and other risk factors [19]. The execution of RCTs is rendered challenging by the observational study’s inherent limitations. Consequently, the investigation of the causal relationship between aspirin use and AF risk faces considerable difficulties.

MR is a sophisticated investigative approach that leverages genetic variables to illuminate causal associations between exposures and outcomes [20]. The utilization of MR facilitates the circumvention of biases by employing instrumental variables (IVs) that serve as indices of exposure to the causality of risk factors associated with a particular disease [21]. However, no research has hitherto been conducted on the application of MR analysis to clarify the causal relationship between aspirin use and AF. Consequently, and to the best of my knowledge, I conducted the first two‐sample MR analysis to ascertain the potential causal relationship between aspirin use and AF risk.

## 2. Method

### 2.1. Study Design

A two‐sample MR analysis design was employed to evaluate the causal relationship between aspirin use and the risk of developing AF, incorporating single nucleotide polymorphisms (SNPs) as IVs. To ensure the greatest precision in the results, it is imperative that three fundamental assumptions are adhered to during the procedure. (1) Genetic instrument variants exhibit a marked correlation with the aspirin use. (2) IVs are not associated with confounders. (3) IVs exert a substantial influence on the risk of AF incidence through the utilization of aspirin, precluding the possibility of influence by alternative processes (Figure 1). This study is reported in accordance with the Strengthening the Reporting of Observational Studies in Epidemiology (STROBE) guidelines, which are designed to ensure the accurate and comprehensive reporting of research findings in MR studies [22].

**Figure 1:**
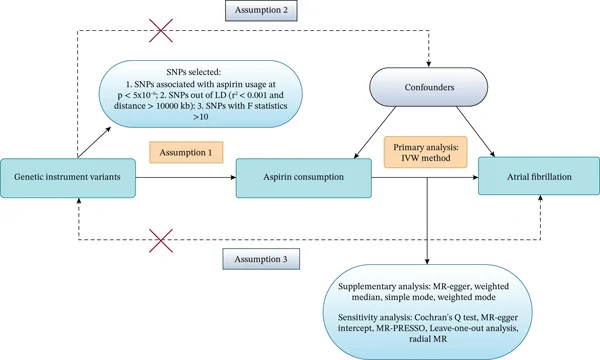
Design flow chart for the MR study. MR assumptions: (1) Assumption 1 suggests that genetic variants correlated with aspirin use and affect atrial fibrillation through aspirin. (2) Assumptions 2 and 3 indicate SNPs are not associated with any confounders and do not influence atrial fibrillation through other ways.

### 2.2. Data Source

I used aspirin use as the exposure and AF incidence as the outcome. Genetic variants associated with aspirin use were identified through GWAS meta‐analyses that were undertaken in individuals of European ancestry (ukb‐b‐8755) with a sample size of 462,933 and (cases = 61,702, controls = 401,231). Summary data from an openly available GWAS study of AF (ebi‐a‐GCST006414) with a sample size of 1,030,836 and (cases = 60,620, controls = 970,216) of European ancestry used as outcome. The data of ukb‐b‐8755 and ebi‐a‐GCST006414 are available at IEU Open GWAS Project (https://gwas.mrcieu.ac.uk/).

### 2.3. IVs Selection

A 2‐stage method was used to identify the IVs. Initially, SNPs demonstrating a strong correlation with aspirin use (*p* < 5*e* − 06) were retrieved. Secondly, independent SNPs exhibiting linkage disequilibrium (LD) (kb = 10,000, *r* ^2^ < 0.001) were retained. IVs strength was assessed by generating the F‐statistic, with a cutoff value of *F* > 10 for the detection of strong IVs. IVs with an F‐statistic of less than 10 were designated as “weak” and consequently excluded from the MR analysis. The data obtained were harmonized to guarantee that the effect of the SNP on exposure and outcome corresponded to the same allele [23]. After the analysis outlined above, the next key step was to confirm that a second MR assumption was met, which had the same effect as introducing the balancing effect of the multivariate analysis. Queries were performed in the PhennoScanner database (http://www.phenoscanner.medschl.cam.ac.uk/) to verify that the selected SNPs were not linked to well‐known confounders such as age, myocardial infarction, coronary heart disease, and hypertension.

### 2.4. Statistical Analysis

The IVW method was used for the primary analysis and together with the Wald ratio of each SNP to the outcome, a pooled causal estimate was obtained [24]. Furthermore, MR‐Egger, weighted median, weighted mode and simple mode methods have been utilized as ancillary analyses. The random‐effects IVW was selected as the primary standard for MR results, with the other methods employed as adjuncts. To evaluate the reliability of the MR estimates, a variety of sensitivity analyses were performed. I used Cochran′s Q test to test for heterogeneity in the data. The intercept in the MR‐Egger method has been shown to serve as a reliable indicator of the potential influence of directional horizontal pleiotropy upon outcomes of MR analysis [25]. The presence of directional pleiotropy in the genetic variants is indicated by a *p* value for the intercept of less than 0.05. A leave‐one‐out sensitivity analysis was carried out to establish the MR result of the residual SNPs after removing each SNP individually. Radial MR was also implemented to identify outliers, and the MR analysis was repeated after removing heterogeneous SNPs. The statistical analyses were conducted using the Two Sample MR and MR PRESSO packages in R software (Version 4.2.1) Statistically significant results were defined as those (*p* < 0.05).

## 3. Results

### 3.1. IVs for MR Analysis

First, 60 SNPs were selected for their robust association with aspirin use (Table S1). Then after removing potential confounders that were positively associated with exposure and the requirement to adjust for SNP effects on both outcome and exposure, 15 SNPs were removed. Finally, I obtained 45 SNPs that met the required criteria and were therefore selected for inclusion in the subsequent MR analysis (Table S2). Three outliers (rs28601761, rs2914648, rs7310615) were removed in the subsequent MR analysis.

### 3.2. MR Analysis

With the IVW method, the evidence obviously supported a causal relationship between aspirin use and AF (OR: 1.96, [95% CI: 1.10–3.47], *p* = 0.021). The weighted median method also indicated a causal association between aspirin use and AF (OR: 2.60 [95% CI: 1.13–5.95, *p* = 0.024]) (Figure 2). The intercept term in the scatter plot represents the average pleiotropic effect across the genetic variants, reflecting the average direct effect of the genetic variants on AF. The presence of a non‐null intercept indicates no presence of directional pleiotropy (Figure 3). Nevertheless, MR‐Egger regression analysis indicated the improbability of such directional pleiotropy biasing the results (MR − Egger intercept = 0.008*p* > 0.05). Conversely, the MR‐Egger regression did not demonstrate a statistically significant causal association between aspirin use and AF (OR: 0.33, 95% CI: 0.05–2.04; *p* = 0.239). MR‐Egger regression is known to have limited statistical power, particularly when the number of IVs is small. In our analysis, only 42 SNPs were available, which may have reduced the power of MR‐Egger to detect causal estimates. Despite the findings concerning the causal relationship between aspirin use and AF being mixed, the primary IVW method implied a causal effect of aspirin use on the risk of AF. Wald ratio of each SNP to the outcome suggested a significant causal association between aspirin use and AF (Figure 4).

**Figure 2:**
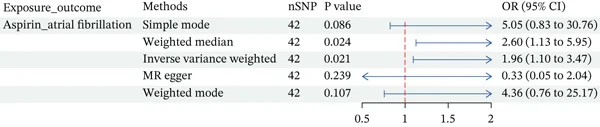
Forest plot of MR results between aspirin use and atrial fibrillation risk.

**Figure 3:**
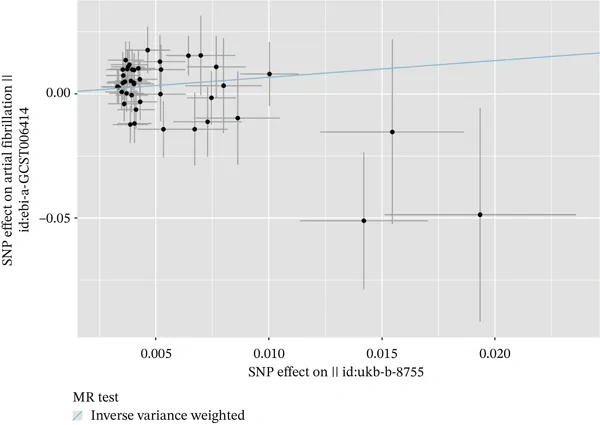
Scatter plot of genetic associations with aspirin and atrial fibrillation risk.

**Figure 4:**
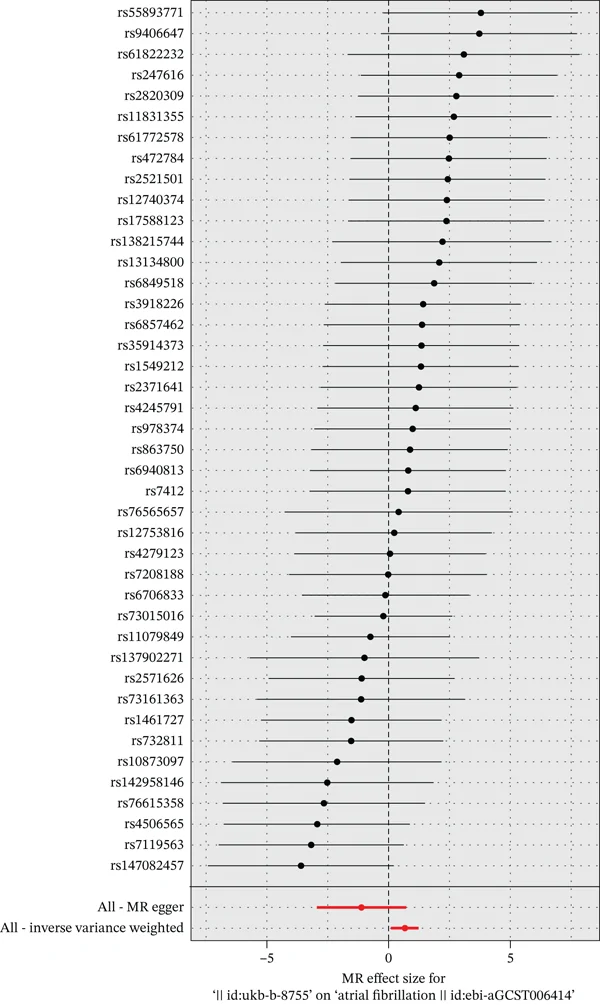
Forest plot of causal effects of SNPs associated with aspirin use on atrial fibrillation risk.

### 3.3. Heterogeneity and Sensitivity

Cochran′s Q test revealed no significant evidence of heterogeneity among the IVs estimates based on individual variants (IVW method, Q statistics = 41.311, *p* = 0.457) suggested high reliability of the MR estimates. The results of the “leave‐one‐out” strategy showed that no single SNP had a significant effect on the IVW estimates (Figure 5), which indicates that the overall causal association was not driven by a specific single SNP. The implementation of radial MR was undertaken with the objective of identifying outliers. This process resulted in the identification of three outliers (rs28601761, rs2914648, rs7310615) and after removing the outliers the causal effect between exposure and outcome was stable (*p* = 0.027) (Figure 6). The initial MR‐PRESSO global test showed a *p* value below 0.05. However, upon the subsequent exclusion of the three outlier SNPs that had been detected by radial MR, a global test using MR‐PRESSO yielded a *p* value greater than 0.05.

**Figure 5:**
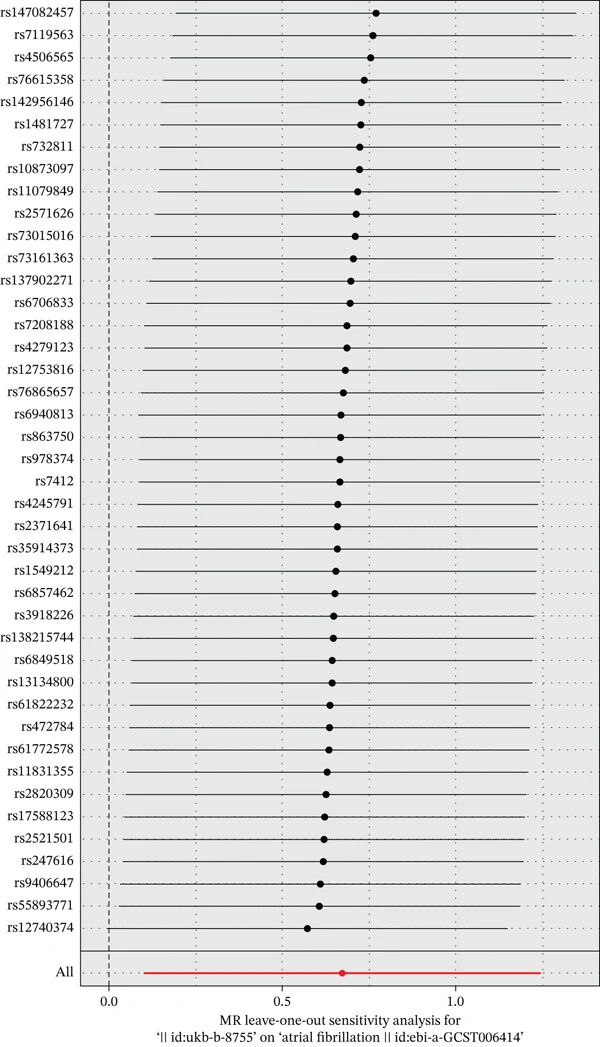
Leave‐one‐out analysis plot of causal effects of individual SNP associated with aspirin on atrial fibrillation risk.

**Figure 6:**
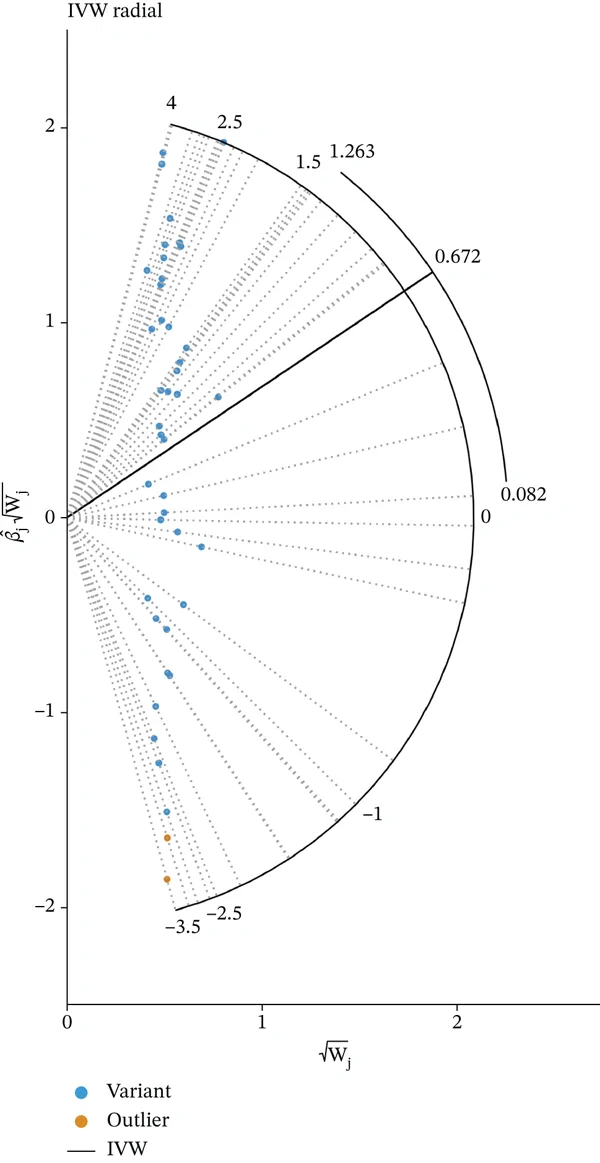
Radial MR analysis plot of causal effects of SNP associated with aspirin use on atrial fibrillation risk.

## 4. Discussion

This study provided the first assessment of the causal relationship between aspirin use and the risk of AF in the European population based on the MR analysis. A causal association with an OR of 1.96 was observed in the mainly genetic predisposition analysis. In further sensitivity analyses, the positive association between the use of aspirin and the risk of AF was strengthened.

Over the past few years, the use of aspirin for the treatment of AF has drawn considerable attention, as the efficacy and safety concerns have been supported by cumulative studies. A recent study determined that adding aspirin is not associated with a reduced risk of ischemic events, even in patients with a high‐risk CHA_2_DS_2_‐VASc score but significantly increases the risk of bleeding in patients with AF undergoing percutaneous coronary intervention (PCI) [26]. The Kailuan cohort study, with 7.2 years of follow‐up, showed that aspirin monotherapy did not benefit patients with AF [27]. Among patients with AF, aspirin use at baseline increased the risk of bleeding and all‐cause mortality, especially in patients who also suffering from coronary heart disease [28]. However, another study concluded that in critically ill patients diagnosed with AF, aspirin use may reduce risk‐adjusted all‐cause mortality in both the short and long term [29]. These discordant conclusions may be attributable to variations in study design and the composition of the enrolled participants. To date, there is limited evidence on aspirin use and the risk of AF, and only one study^19^ that did not support a causal relationship between AF and aspirin use after adjusting for other confounding risk factors. However, the study has limitations, including the lack of systematic electrocardiogram monitoring to objectively detect AF and the use of an algorithm to categorize AF. Therefore, future population studies are required to further explore the effect of aspirin on AF incidence. In recent times, an increasing number of studies have focused on the side effects of aspirin use. Li et al. found that aspirin use increased erectile dysfunction risk (OR = 20.90 [95% CI 2.08, 2.10e+2]) by MR analysis [30]. Liu determined that the utilization of aspirin resulted in a substantial augmentation in lumbar spine bone mineral density (OR = 4.66 [95% CI 1.37, 15.91]) [31]. The present study for first time determined that aspirin use increase AF incidence risk by MR analysis.

It has been established that aspirin may inhibit the production of prostaglandins, consequently resulting in vasodilation and gastrointestinal injury [32]. In addition, it is recognized that left atrial fibrosis may be an important factor and predictor in the onset and development of AF [33]. Prostaglandin I2 (PGI2) is produced by the metabolism of arachidonic acid by cyclooxygenase‐2 (COX‐2) and PGI synthase (PTGIS) following activation by various exogenous physical and chemical irritants [34]. It is well established that PGI2 functions as a significant antifibrotic factor, inhibiting the synthesis of the extracellular matrix (ECM) [35]. Zhang et al. recently reported a decrease in the PGI2 content of patients with AF and mice models. Furthermore, they also indicated that increased PGI2 could inhibit angiotensin II (Ang II) levels [36]. Ang II has been demonstrated to induce the development of AF by transforming growth factor beta‐1 (TGF‐*β*1) and peroxisome proliferator‐activated receptor *α* (PPAR*α*) [37, 38]. Thus, aspirin may promote the onset of AF by inhibiting PGI2 and subsequently upregulating Ang II, which can result in atrial fibrosis. In a large‐scale RCT, aspirin significantly increased anemia incidence risk (HR = 1.20 [95% CI 1.12, 1.29]) [39]. Interestingly, evidence from a cohort study indicated that anemia increased the incidence of AF [40]. Moreover, patients suffering from anemia appear to be more susceptible to the clinical re‐occurrence of AF [41]. Ferroptosis is a distinct form of regulated cell death that is characterized by iron‐dependent lipid peroxidation. This process is intricately orchestrated by integrated oxidation and antioxidant systems, resulting in the controlled demise of cells [42]. It is found that aspirin may involve in inducing ferroptosis in carcinoma [43]. Dai et al. suggest that inhibition of ferroptosis reduces susceptibility to AF induced by chronic alcohol abuse [44]. Myocardial ferroptosis contributes to atrial structural remodeling and increases oxidative stress, thereby promoting AF [45]. Consequently, aspirin also has the potential to induce the occurrence of AF by modulating anemia and myocardial ferroptosis.

It is imperative to emphasize that genetic variants are established over the course of an organism′s life, and alleles are distributed randomly. Consequently, the findings of this study represent the lifetime risk of AF associated with aspirin use in the population and may mitigate bias due to confounding and reverse causality. Additionally, data from an extensive GWAS of risk factors was utilized to minimize confounding effects within specific population strata, with most of the data derived from cohorts of European descent. The investigation was conducted based on three key assumptions underpinning the MR analysis. The power of the findings was maintained by excluding SNPs with potential effects, outliers and pleiotropic effects, thereby minimizing bias in the assessment of causal effects.

The following limitations must be considered when interpreting the results. (1) The applicability of the results to other populations may be constrained because my analysis was only of European ancestry. (2) Although measures were adopted to mitigate pleiotropy, it cannot be fully eliminated in MR studies. Unidentified pleiotropic pathways and residual confounding between the exposure and outcome may therefore persist—several instrumental variants are located within loci associated with cardiovascular and metabolic traits—and these could introduce bias into our estimates. (3) Given that the exposure and outcome datasets were both derived from the UK Biobank, sample overlap cannot be fully excluded. This may introduce bias and inflate the risk of false‐positive findings, and independent replication is therefore warranted. (4) Because of constraints on data availability, this study examined aspirin use without distinguishing drug targets or dose. Consequently, the findings should be interpreted in this context, and the relationship between aspirin and AF requires further investigation. (5) The exposure GWAS reflects genetic liability to aspirin use rather than the direct biological effect of aspirin itself. Accordingly, the present study suggests a potential causal association between aspirin use and the incidence risk of AF. (6) To confirm and extend these observations, future large‐scale, rigorously designed, population‐based studies with long‐term follow‐up will be required.

## 5. Conclusion

In conclusion, to my best knowledge, this is the first MR study to examine the causal association between aspirin use and AF. My findings suggest a potential causal relationship whereby aspirin use may be associated with an increased risk of AF. However, these results should be interpreted with caution in light of the inherent limitations of the MR design, including the possibility of undetected residual pleiotropy, potential sample overlap between the exposure and outcome datasets, and the inability to account for drug targets or dose–response effects. Nevertheless, this study provides preliminary evidence that may inform future research and clinical considerations regarding aspirin use and AF risk. Well‐designed, large‐scale prospective studies with long‐term follow‐up and detailed exposure assessment are warranted to confirm and extend these observations.

## Author Contributions

Gang Fan conceptualized and supervised the study, conducted the statistical analyses, and drafted the manuscript.

## Funding

No funding was received for this manuscript.

## Ethics Statement

The author has nothing to report.

## Consent

The author has nothing to report.

## Conflicts of Interest

The author declares no conflicts of interest.

## Supporting Information

Additional supporting information can be found online in the Supporting Information section.

## Supporting information


**Supporting Information 1** STROBE‐MR‐checklist.

**Supporting Information 2** Table S1: Selected IVs (before clumping) for the study.

**Supporting Information 3** Table S2: Selected IVs for the study.

## Data Availability

The data that support the findings of this study are available from the corresponding author upon reasonable request.
