# Supplementary material for: Aspirin Use May Increase Atrial Fibrillation Risk: A Mendelian Randomization Analysis
Source: Int J Genomics. 2026 Nov 15;2026:6124554. doi: 10.1155/ijog/6124554 (PMC13770272; doi:10.1155/ijog/6124554)
Supplement: Supplementary file 1 [file IJOG-2026-6124554-s001.docx]

**STROBE-MR checklist of recommended items to address in reports of Mendelian randomization studies**

| **Item No.** | **Section** | **Checklist item** | **Page No.** | **Relevant text from manuscript** |
| --- | --- | --- | --- | --- |
| 1 | **TITLE and ABSTRACT** | Indicate Mendelian randomization (MR) as the study’s design in the title and/or the abstract if that is a main purpose of the study | **1** | Aspirin use may increase atrial fibrillation risk: a Mendelian randomization analysis |
|  | **INTRODUCTION** |  |  |  |
| 2 | **Background** | Explain the scientific background and rationale for the reported study. What is the exposure? Is a potential causal relationship between exposure and outcome plausible? Justify why MR is a helpful method to address the study question | 1 | Introduction section |
| 3 | **Objectives** | State specific objectives clearly, including pre-specified causal hypotheses (if any). State that MR is a method that, under specific assumptions, intends to estimate causal effects | 1 | The relationship between aspirin consumption and atrial fibrillation (AF) remains uncertain. This study aimed to determine the causal association of aspirin consumption and AF risk by Mendelian randomization (MR) analysis. |
|  | **METHODS** |  |  |  |
| 4 | **Study design and data sources** | Present key elements of the study design early in the article. Consider including a table listing sources of data for all phases of the study. For each data source contributing to the analysis, describe the following: |  |  |
|  | a) | Setting: Describe the study design and the underlying population, if possible. Describe the setting, locations, and relevant dates, including periods of recruitment, exposure, follow-up, and data collection, when available. | 1 | Single nucleotide polymorphisms (SNPs) for aspirin consumption and AF were obtained from genome-wide association studies (GWAS). I applied inverse variance weighted (IVW) as the primary MR analysis method. In addition, I performed Cochran's Q, MR-Egger's intercept, leave-one-out, MR-PRESSO, and Radial MR to evaluate heterogeneity, horizontal pleiotropy, sensitivity, and reliability of the results. |
|  | b) | Participants: Give the eligibility criteria, and the sources and methods of selection of participants. Report the sample size, and whether any power or sample size calculations were carried out prior to the main analysis | 4 | IV Selection |
|  | c) | Describe measurement, quality control and selection of genetic variants | 4 | Statistical section |
|  | d) | For each exposure, outcome, and other relevant variables, describe methods of assessment and diagnostic criteria for diseases | 4 | Aspirin consumption and atrial fibrillation incidence |
|  | e) | Provide details of ethics committee approval and participant informed consent, if relevant | 8 | This study did not involve direct interaction with human participants or access to identifiable human data and was conducted using publicly available data. |
| 5 | **Assumptions** | Explicitly state the three core IV assumptions for the main analysis (relevance, independence and exclusion restriction) as well assumptions for any additional or sensitivity analysis | 3 | Study design |
| 6 | **Statistical methods: main analysis** | Describe statistical methods and statistics used |  |  |
|  | a) | Describe how quantitative variables were handled in the analyses (i.e., scale, units, model) | 4 | Statistical section |
|  | b) | Describe how genetic variants were handled in the analyses and, if applicable, how their weights were selected | 4 | IV Selection |
|  | c) | Describe the MR estimator (e.g. two-stage least squares, Wald ratio) and related statistics. Detail the included covariates and, in case of two-sample MR, whether the same covariate set was used for adjustment in the two samples |  | The present study used inverse-variance weighted (IVW) as the primary analysis, which combined with Wald ratio of each SNP on the outcome and gained a pooled causal estimate |
|  | d) | Explain how missing data were addressed |  | This study did not used the proxy and the missing data were excluded |
|  | e) | If applicable, indicate how multiple testing was addressed |  |  |
| 7 | **Assessment of assumptions** | Describe any methods or prior knowledge used to assess the assumptions or justify their validity |  | Aspirin consumption may increase atrial fibrillation onset |
| 8 | **Sensitivity analyses and additional analyses** | Describe any sensitivity analyses or additional analyses performed (e.g. comparison of effect estimates from different approaches, independent replication, bias analytic techniques, validation of instruments, simulations) | 4 | Statistical section |
| 9 | **Software and pre-registration** |  |  |  |
|  | a) | Name statistical software and package(s), including version and settings used | 6 | R software (version 4.2.1) |
|  | b) | State whether the study protocol and details were pre-registered (as well as when and where) |  | no |
|  | **RESULTS** |  |  |  |
| 10 | **Descriptive data** |  |  |  |
|  | a) | Report the numbers of individuals at each stage of included studies and reasons for exclusion. Consider use of a flow diagram | 3 | Data source section |
|  | b) | Report summary statistics for phenotypic exposure(s), outcome(s), and other relevant variables (e.g. means, SDs, proportions) |  | OR and 95%CI |
|  | c) | If the data sources include meta-analyses of previous studies, provide the assessments of heterogeneity across these studies |  | No |
|  | d) | For two-sample MR:  i.  Provide justification of the similarity of the genetic variant-exposure associations between the exposure and outcome samples  ii.  Provide information on the number of individuals who overlap between the exposure and outcome studies |  | The exposure and outcome data from different database, so there is no overlap of individuals involved |
| 11 | **Main results** |  |  |  |
|  | a) | Report the associations between genetic variant and exposure, and between genetic variant and outcome, preferably on an interpretable scale | 5 | Results section |
|  | b) | Report MR estimates of the relationship between exposure and outcome, and the measures of uncertainty from the MR analysis, on an interpretable scale, such as odds ratio or relative risk per SD difference |  |  |
|  | c) | If relevant, consider translating estimates of relative risk into absolute risk for a meaningful time period |  |  |
|  | d) | Consider plots to visualize results (e.g. forest plot, scatterplot of associations between genetic variants and outcome versus between genetic variants and exposure) |  | Added in the manuscript |
| 12 | **Assessment of assumptions** |  |  |  |
|  | a) | Report the assessment of the validity of the assumptions |  | IVW, Cochran's Q, MR-Egger's intercept, leave-one-out, MR-PRESSO, and Radial MR were used to verify the assumptions. |
|  | b) | Report any additional statistics (e.g., assessments of heterogeneity across genetic variants, such as *I^2^*, Q statistic or E-value) |  |  |
| 13 | **Sensitivity analyses and additional analyses** |  |  |  |
|  | a) | Report any sensitivity analyses to assess the robustness of the main results to violations of the assumptions |  | The sensitivity analysis confirmed the robustness of the MR findings [horizontal pleiotropy (MR-Egger, *p>*0.05)]. No heterogeneity was observed among the SNPs, as indicated by Cochran Q test (*p>*0.05). |
|  | b) | Report results from other sensitivity analyses or additional analyses |  | The sensitivity analysis confirmed the robustness of the MR findings [horizontal pleiotropy (MR-Egger, *p>*0.05)]. No heterogeneity was observed among the SNPs, as indicated by Cochran Q test (*p>*0.05). |
|  | c) | Report any assessment of direction of causal relationship (e.g., bidirectional MR) |  | Aspirin consumption increases the risk of atrial fibrillation |
|  | d) | When relevant, report and compare with estimates from non-MR analyses |  |  |
|  | e) | Consider additional plots to visualize results (e.g., leave-one-out analyses) |  | Already added in the manuscript |
|  | **DISCUSSION** |  |  |  |
| 14 | **Key results** | Summarize key results with reference to study objectives |  | The findings of this MR imply that there is a positive causal relationship between aspirin consumption and the onset of AF. Future long-term large-scale studies are needed to further verify the results. |
| 15 | **Limitations** | Discuss limitations of the study, taking into account the validity of the IV assumptions, other sources of potential bias, and imprecision. Discuss both direction and magnitude of any potential bias and any efforts to address them |  | The following limitations must be considered when interpreting the results. (1) The applicability of the results to other populations may be constrained because my analysis was only of European ancestry. (2) **Although measures were adopted to mitigate pleiotropy, it cannot be fully eliminated in MR studies. Unidentified pleiotropic pathways and residual confounding between the exposure and outcome may therefore persist—several instrumental variants are located within loci associated with cardiovascular and metabolic traits—and these could introduce bias into our estimates.** (3) Given that the exposure and outcome datasets were both derived from the UK Biobank, sample overlap cannot be excluded. This may introduce bias and inflate the risk of false-positive findings, and independent replication is therefore warranted. **(4) Because of constraints on data availability, this study examined aspirin use without distinguishing drug targets or dose. Consequently, the findings should be interpreted in this context, and the relationship between aspirin and atrial fibrillation requires further investigation.** (5) **The exposure GWAS reflects genetic liability to aspirin use rather than the direct biological effect of aspirin itself. Accordingly, the present study suggests a potential causal association between aspirin use and the incidence risk of atrial fibrillation.** (6) To **confirm and extend these observations, future large-scale, rigorously designed, population-based studies with long-term follow-up will be required.** |
| 16 | **Interpretation** |  |  |  |
|  | a) | Meaning: Give a cautious overall interpretation of results in the context of their limitations and in comparison with other studies |  | This Mendelian randomization study provides evidence suggesting a potential causal association between aspirin use and an increased risk of atrial fibrillation. However, given the inherent limitations of the MR design, these findings should be interpreted with caution. Future large-scale studies with long-term follow-up are warranted to confirm and extend these observations. |
|  | b) | Mechanism: Discuss underlying biological mechanisms that could drive a potential causal relationship between the investigated exposure and the outcome, and whether the gene-environment equivalence assumption is reasonable. Use causal language carefully, clarifying that IV estimates may provide causal effects only under certain assumptions |  | Aspirin may promote the onset of AF by inhibiting PGI2 and subsequently upregulating Ang II, which can result in atrial fibrosis |
|  | c) | Clinical relevance: Discuss whether the results have clinical or public policy relevance, and to what extent they inform effect sizes of possible interventions |  | Our findings stress the significance of paying more attention to aspirin consumption and atrial fibrillation onset. |
| 17 | **Generalizability** | Discuss the generalizability of the study results (a) to other populations, (b) across other exposure periods/timings, and (c) across other levels of exposure | 11 | This study is the first to suggest a positive association between aspirin consumption and atrial fibrillation risk. Our findings stress the significance of paying more attention to aspirin consumption in clinical settings. |
|  | **OTHER INFORMATION** |  |  |  |
| 18 | **Funding** | Describe sources of funding and the role of funders in the present study and, if applicable, sources of funding for the databases and original study or studies on which the present study is based |  | Not available |
| 19 | **Data and data sharing** | Provide the data used to perform all analyses or report where and how the data can be accessed, and reference these sources in the article. Provide the statistical code needed to reproduce the results in the article, or report whether the code is publicly accessible and if so, where |  | Data can be accessed from the corresponding author on reasonable purpose |
| 20 | **Conflicts of Interest** | All authors should declare all potential conflicts of interest |  | The authors declare no competing interests |

This checklist is copyrighted by the Equator Network under the Creative Commons Attribution 3.0 Unported (CC BY 3.0) license.

1.	Skrivankova VW, Richmond RC, Woolf BAR, Yarmolinsky J, Davies NM, Swanson SA, et al. Strengthening the Reporting of Observational Studies in Epidemiology using Mendelian Randomization (STROBE-MR) Statement. JAMA. 2021;under review.

2.	Skrivankova VW, Richmond RC, Woolf BAR, Davies NM, Swanson SA, VanderWeele TJ, et al. Strengthening the Reporting of Observational Studies in Epidemiology using Mendelian Randomisation (STROBE-MR): Explanation and Elaboration. BMJ. 2021;375:n2233.
